# Puerperal Sepsis; Its Prevention and Cure1Read by title at the eighty-seventh annual meeting of the Medical Society of the State of New York, February, 1893, and published in the *Annals of Gynecology and Pediatry*, April, 1893.

**Published:** 1893-08

**Authors:** William Warren Potter

**Affiliations:** Buffalo, N. Y.; Fellow of the American Association of Obstetricians and Gynecologists; Examiner in Obstetrics, New York State Medical Examining and Licensing Board; 284 Franklin Street


### Original Communications.

### PUERPERAL SEPSIS; ITS PREVENTION AND CURE.[1]

By WILLIAM WARREN POTTER, M. D., Buffalo, N. Y.

Fellow of the American Association of Obstetricians and Gynecologists; Examiner in Obstetrics, New York State Medical Examining and Licensing Board.

### I. INTRODUCTION.

Among the many serious questions urged upon the attention of physicians at the present time, none have higher claims to their thoughtful consideration than such as pertain to the prevention of disease. If this be true in its larger sense when applied to the entire field of preventive medicine, it is even more emphatically true when we are called upon to consider those delicate and serious questions that especially concern the lying-in chamber. Here, if anywhere, a man is weighted with responsibilities of a momentous nature, even such as he is called upon to sustain in no other social, professional, or individual capacity. They are often thrust upon him in the middle of the night, when he is least fitted for clear thought or resolute action; hence, he must, by frequent discussion and much study, keep his mental armamentarium always burnished and ready for use at a moment's notice. The custom is, I fear, among some—I had almost said many—physicians to assume these responsibilities in a light-hearted, easy-going manner that occasionally approaches even to triviality. In my view, there is no more solemn office to be performed by a human being than in assisting at the delivery of one of his species. To preside over such an event, and to conduct it to successful issue, without permitting danger to mother or infant through neglect or oversight, is to accomplish something that benefits humanity, prolongs life, and adds to the sum of human happiness.

### II. OF PREVENTION—(*a*) AS RELATES TO THE PHYSICIAN.

In order to perform this duty properly, that is, in a manner to obtain its greatest results, it is essential, *inter alia*, that the

1. Read by title at the eighty-seventh annual meeting of the Medical Society of the State of New York, February, 1893, and published in the *Annals of Gynecology and Pediatry*, April, 1893.

obstetrician should be trained in habits of personal cleanliness beyond those of the ordinary man; and even, if I may be permitted to say it, beyond those of the average physician. If a man is not scrupulous with reference to the care of his person, how can he be expected to enforce a technical cleanliness in the lying-in chamber? A man who holds himself up to the community as an obstetrician should be a model in cleanliness of body as well as in neatness of attire. He should take frequent baths, wear clean underclothing and linen, but especially should he keep his hair and beard closely trimmed and his finger-nails short and clean. If the physician himself is careless of his personal garb and cleanliness, he is placed at a disadvantage with reference to enforcing the observance of these essentials in others. He, then, is a commander-in-chief, who supervises everything with reference to the parturient chamber, and enforces his mandates with the discipline of a martinet in everything that serves to prevent the approach of that stealthy, thieving, sneaking but all-powerful enemy, dirt.

(*b*) AS RELATES TO THE CONFINEMENT ROOM AND NURSE.

The lying-in chamber should receive his personal supervision in reference to its simplicity, cleanliness, and freedom from hangings or draperies. If the room is a humble one, where luxuries are absent and even necessities deficient, there still should be given the same careful attention to cleanliness that prevails in the well-appointed home. A free use of soap and water and the whitewash brush will do much to provide against contamination and disease. The nurse, too, who is to perform her offices in the lying-in room, must be a woman of neatness in garb and cleanliness of person beyond the average of womankind. She must be trained in these habits until they become her second nature. She must not be afraid to attack dirt wherever she sees it, nor unwilling to act as a sentinel or detective with reference to that masterful foe of the lying-in chamber.

(*c*) AS RELATES TO THE DELIVERY BED.

One of the important duties that a physician must perform in arranging for the ordeal that awaits his patient is to look to the proper preparation of the bed. If it is important that the clothing of the patient be clean, it is still more essential that her bed should be the very quintessence of cleanliness. The mattress should be inspected with reference to its freshness as well as its former use, and all the linens and other dressings of the bed should receive his most careful scrutiny in this regard. If the station of the patient does not admit of more than the bare necessities of the bed-chamber, then a clean bed-tick, filled with sweet, clean straw, covered with a blanket and an impervious dressing of some kind, over which is to be laid a folded sheet, furnishes all that is necessary for the comfort, and even convenience, of any patient. If I could have my own way, I would allow no woman, whether of high or low degree, to be confined upon anything but a straw bed which had been prepared especially for the occasion. I lay great stress upon the preparation of the bed. Neglect in this regard may be a source of bitter sorrow, whereas a little timely and considerate attention to it beforehand may serve to prevent disaster.

If, perchance, a woman has come to her old home to give birth to her first child, it will not answer to allow her to occupy the bed upon which, a few months ago, her sister lay grievously ill with scarlet fever, or on which her brother died with diphtheria; nor will it be proper on this important occasion to permit her confinement in the bed where her father was treated for a compound fracture of the tibia and fibula; or that on which her husband lay during long and weary weeks while struggling with typhoid fever. It may be the favorite family bed for all such trying occasions, but there is a particular reason why we must discard it this time. I have no doubt that many a case of puerperal sepsis is traceable to the use of such a bed during delivery. Hence, I say, it would be better to establish a hard and fast rule that every patient, no matter how rich, no matter how poor, should be compelled to occupy a new straw bed during her confinement. This ought to be exchanged subsequently for a clean mattress, to be occupied during puerperal convalescence.

### (*d*) AS RELATES TO THE PARTURIENT.

The patient herself should be properly prepared for the ordeal that awaits her. She should have a warm bath as near the onset of labor as may be, and with the oncoming of pain should be thoroughly bathed, antiseptically, over the abdomen, in the groins, on the vulva, hips and thighs. After the bladder and rectum have been emptied, this should be repeated, when the rectum and vagina should receive warm lavements. Then, in a clean night-gown and light woolen stockings, she may be considered ready to occupy the delivery bed. The conduct of the labor should continue to its end on the lines of absolute cleanliness, with few digital examinations, and complete delivery of the secundines.

(*e*) AS RELATES TO THE REPAIR OF LESIONS.

Finally, I need scarcely remark that one available means of great importance in preventing puerperal sepsis abides in the prompt repair of all rents in the perineum. This removes an important complication at once, as a large absorbent surface is thus readily disposed of. There is no question in the mind of any well-trained physician today that unrepaired perineal rents are a septic menace, or that in many instances sepsis has invaded the system through this channel. There is sufficient reason in this danger alone to warrant immediate repair without discussing the other important fact not germane to this paper, namely, that it is essential to a woman's health and comfort, as well as to those of her consort, to possess a sound perineum. In some instances, likewise, it is advisable to repair cervical tears immediately, especially when they are extensive enough to cause much hemorrhage, or threaten to prolong the puerperal period by delaying involution or provoking other serious conditions.

III. OF CURE.

If thus far I have said little or nothing with reference to the employment of antiseptics or chemical solutions, this has not been an oversight on my part, but because I consider these of far less importance in the prevention of puerperal sepsis than a strict adherence to the principles of asepsis in the preparation for, as well as in the management of, labor. I have an impression that if we could *properly* apply the rules of cleanliness in every case, there would be no sepsis, and, hence, no puerperal septicemia or child-bed fever, as it was called in the olden time. It is possible, when every principle of mechanics known to science is properly applied, and when human judgment is kept clear and free to apply it, to prevent any and every form of railway accident that threatens or destroys life or limb. When we come to consider the great number of people who travel, we must admit that the number killed is very small, but still it is yet far too great to satisfy that tender regard for human life which should everywhere prevail among mankind. So, too, do I believe that every case of puerperal sepsis could be prevented, provided human judgment was perfect in its application of the principles of asepsis or cleanliness. Chemical solutions are valuable, and must be resorted to by the obstetrician to keep his hands and instruments clean, and also for the purpose of rendering aseptic the linen and other cloths that are used around and about the parturient woman. But, if the proper rule of cleanliness is observed—in other words, if everything about her is rendered aseptic—there will be no necessity for the employment of antiseptics in an ordinary labor otherwise than as just indicated.

In operative midwifery, either manual or instrumental, there is always danger that pathogenic germs may be carried into the genital tract by the instruments or hands of the operator. Then it is that antiseptics become necessary to clean out and wash away the dirt, or to neutralize the effects of such as has been carried into the absorbent, surgical uterine cavity.

Since, therefore, in spite of all known ways to prevent such direful calamity, puerperal sepsis will now and then come—just as in spite of all human precaution railway accidents will now and then happen—we must be prepared to cure, if possible, the former, just as railway corporations must be prepared to mitigate the suffering and clean up the wreckage entailed by the latter. I shall take but a few moments of your time in the consideration of this part of my subject. If there is invasion of the genital tract for the purpose of promoting delivery, either by hands or instruments, then we must be wary lest secondary conditions follow, that further complicate or hazard the patient's chances of recovery. Intrauterine irrigation must be performed in every case where such aids to delivery have been employed, and when done, it must be carried out in the most complete modern surgical manner. The woman must be placed upon a proper table, in a good light, and the fluid—either sterilized water, or water that has been boiled and contains an adequate germicide—must be allowed to flow into and out of the womb, in an unobstructed manner, until it runs clear and is absolutely clean. There must be no detritus left behind; no nidus for the propagation of the germs of infection. The mistake has been made of applying intrauterine irrigation inadequately, either as to the method of employing it or as to the material used. Either form of error is sufficient to bring failure, or to render it useless or nugatory. I shall not here enter into technical and minute details, for this has already amply been done by Dr. L. S. McMurtry, of Louisville, honorary member of this society,[1] but I simply offer these general suggestions as to the indispensabilities of the case, leaving the minutiæ to be supplied by the ingenuity of the physician when the occasion and necessity are presented. There are certain things that must be done in such an emergency. The woman must be made clean from the fungus uteri to the ostium vaginæ, and she must be kept so ; futhermore, it must be done in a manner and with a material that will do her no harm. This is the whole sum and substance of the case, and we may as well, first as last, settle down upon some fixed and determined principles in regard to this point.

But when, in spite of the prompt and persistent employment of intrauterine irrigation, associated with the use of the curette when indicated, as well as other concomitant preventive measures, puerperal sepsis continues to advance to a stage of suppuration and abscess, what shall be done ? Without entering into a full discussion of this interesting subject, I shall content myself, on this occasion, with the remark that, in such a case, and in the light of our present knowledge, a prompt abdominal section, by a competent man, is not only entirely justifiable, but is the most intelligent step to be taken in the treatment. All pus accumulations should be cleaned out, irrigation thoroughly employed, and drainage used. In case the uterine body has itself become saturated with sepsis, the propriety of its removal, together with its appendages, cannot be doubtful. The record of recoveries from such desperate conditions is sufficient to justify its employment as a necessary measure in such a direful class of cases. It needs no argument to prove to this audience that a simple abdominal section, with irrigation and drainage, would not be adequate to cure, providing the uterus and its appendages were thoroughly saturated with pus. It may be a more difficult question to decide as to just what ought to be done in cases where sepsis still persists in its manifestations after abdominal section, intraperitoneal irrigation, and adequate drainage. But it would appear rational to reopen the abdomen in such cases, and excise the putrescent uterus, provided, of course, there is sufficient remaining strength in the patient to warrant the procedure.

### GENERAL REMARKS.

In the consideration of this subject, I have, designedly, only here and there touched upon salient features for the purpose of

1. Trans. Am. Asso. Obst. and Gynec., vol. iv., 1891, p. 26, *et seq.*

covering as much ground as I could in the short time allowed There is scarcely one of the subdivisions of this subject that would not admit of an exhaustive paper. But, in order to command the respect, not to say attention, of audiences in these days, writers must evermore be brief. My purpose has been to make a plea for cleaner and more considerate obstetrics. I would do away with all haste, approach each case with due consideration for its best interests, and a careful preparation to meet its complications.

A few years ago it was considered next to suicidal for a woman to enter a maternity hospital for confinement. All this has changed, and nowadays a maternity is regarded on all hands, when properly conducted, as the safest and best place in which a woman can be delivered. When these large institutions are presenting a series of one thousand consecutive cases without a death, embracing in many instances patients who have come within their doors almost moribund, embracing also patients upon whom it has been necessary to perform a Porro or other Cesarean section, I say, in view of all this record, it becomes the obstetrician who conducts a private practice to beware, lest the maternity physician strips him of his laurels. The abdominal surgeon takes pride in presenting a year's record of a hundred consecutive cases without a death, but he is only enabled to do this by the utmost precision, careful preparation of his patient, and a strict adherence to the doctrines of the gospel of cleanliness. When the obstetrician engaged in general practice can also present a record of a thousand consecutive cases without a death, or without serious, prolonged or grievous maiming to his patient, he will then stand in the front rank and deserve to have his name enrolled alongside of the maternity physician and the abdominal surgeon as having done, perhaps, more than either to improve the condition of his fellow-men.

I have only suggested in this paper some of the simpler ways through which this may be achieved. They are such as ought to commend themselves to every thinking man, and were known to all of you just as well before their enumeration as afterward; but, as I have suggested, there are reasons which lead me to believe that it is essential to review this subject now and again, for it is only through the Grant plan of continual hammering that triumphant victory can be wrung from a threatened humiliating defeat. In justification of what I have just now said, permit me, in conclusion, to remark that not long ago it was my privilege to read a paper on a subject allied tc this, which was published in the *Phila-*

*delphia Medical News.* The editor of the Kansas City *Medical Record,* in the August issue of his journal, saw fit to comment upon it as follows :

Dr. William Warren Potter, of Buffalo, appears in the *Medical News* with a paper with this caption, Asepsis and Antisepsis as Applied in the Lying-in Chamber, in which the following paragraphs appear :

Here are a few simple propositions about which there can be, or at least ought to be, no dispute : 1. Let us begin by making the patient as nearly clean as it is possible for soap and water to accomplish. 2. Let her, prior to the beginning of labor, have an immersion bath daily for several days, and with the first manifestations of pains let her abdomen and genitalia be rendered absolutely aseptic by the further application of germicides in solution, adequate to accomplish the desired end. 3. Let her have a warm vaginal douche, rendered aseptic. 4. Let the lower bowel be thoroughly evacuated by copious lavements of hot water prior to the vaginal bath. 5. Let her bedding be made as pure and clean as careful laundrying can make it. 6. Let her clothing be made equally clean in like manner. 7. Let there be a number of clean bichloride napkins placed in readiness for use. If all of these injunctions are rigidly enforced, we have done much to lay the foundation for a physiologic labor.

After all this careful preparation of the patient and her surroundings, we have not, however, done enough. The physician and all the attendants must be rendered as scrupulously clean and aseptic as the patient herself, else all the previous preparation has been in vain. The nurse must be a woman of absolute cleanliness, both in the care of her person and her clothing, and she must be especially trained in the habit of keeping her hands clean. The physician must be trained in all the details of aseptic and antiseptic principles, and must enforce his rules as rigidly upon himself as he does upon his patient and her nurse.

Now, while this is all perfectly correct practice in Buffalo or other large cities where so many septic conditions abound, it is not called for in the rural districts. Especially should every precaution be observed at maternity or other hospitals where women are to be confined. Dr. Potter deplores the fact that practitioners in the rural districts cannot carry out every injunction given above, and seems to be impressed with the idea that they must, therefore, have bad results.

We desire to remark for the benefit of Dr. Potter that nine out of every ten country practitioners of twenty or thirty years' practice never see a case of puerperal sepsis, in spite of the fact that many women in the country, particularly the far west, are confined under the most adverse conditions, many of them surrounded by filth, and living in houses not good enough for horses, where the kitchen, dining-room, drawing-room, parlor, bed-room and dog-kennel are all one and the same apartment. Furthermore, they may not even receive the advantage of a physician, but are delivered by some filthy old midwife, and left for days surrounded by all the dirty clothing employed during confinement. Yet these parturient women do nicely, and the so-called puerperal fever, if not unknown to them, very rarely occurs. The truth is, the pathogenic germs are rarely found in the country; therefore, the care of the patient in crowded cities is not absolutely necessary in country places.

If Dr. Potter had made himself familiar with the facts, he would not have made use of such suggestions as appear in the following paragraph:

As a final thought, permit me to observe that with greater care given by the general practitioner in the remote portions of the country and away from the large centers, as well as in the populous cities, it is my opinion that so-called puerperal fever may be eliminated as a factor of constant menace in the parturient chamber, and that ophthalmia neonatorum may be absolutely and entirely prevented.

I need not detain you with a rejoinder to this ill-considered criticism. I read it to you as a justification, in some sense, for appropriating so much of your valuable time in cultivating ground that has been well plowed and harrowed before.

When physicians can be found—and especially editors, who ought to be leaders in sound medical thought and opinion—to take the position of our Kansas City friend, then, I repeat, there is still room for much agitaiion on this subject.

It will be observed that I have not advocated a complicated technique, with never-ending formula for chemical solutions, and complicated rules for their use that nobody could be expected to obey; but a simple plan that anybody can carry out in the tenement house, cottage, or mansion. But I detain you too long.

In conclusion, I beg to submit the following summary, embracing the principal points for discussion evolved in this paper:

1.—Obstetric engagements once accepted should be faithfully fulfilled, no matter how awkwardly they fit. Apply the same rule of cleanliness to rich and poor alike. Decline service when this cannot be done. Human life is too precious to jeopardize it by slip-shod, half-hearted, or indifferent service.

2.—The physician should be a model of cleanliness in body and clothing, and should insist upon the observance of similar conditions by all persons in and about the lying-in chamber.

3.—The delivery room, whether in hovel or palace, court, alley or avenue, should be simple in its furniture and hangings, and be cleaned with soap, water, and whitewash (if possible to use the latter) immediately before occupancy by the puerpera.

4.—The delivery bed should consist of a new tick filled with sweet and clean straw, covered with a blanket, impervious dressing and a folded sheet, with other clean covering to be allowed, according to season. Exceptions to this simple bed should be as few as possible, and in no event should a bed be substituted that has been used by the sick, or that is not beyond even a suspicion of infection.

5.—The patient should be specially prepared for delivery by baths and enemata, vaginal douches, and clean clothing; and labor should be conducted on the lines of absolute cleanliness, with few digital examinations, and a complete delivery of the secundines.

6.—Lesions of the genital tract should receive careful attention; rents of the perineum should be repaired, and so, too, in some instances should tears of the cervix.

7.—Antiseptic solutions containing a germicide should be used for cleaning the hands and instruments of the operator. Intrauterine irrigation with sterilized water should be carefully employed after operative midwifery, either manual or instrumental.

8.—Finally, if sepsis proceed to suppuration and abscess, the abdomen should be opened, pus cavities emptied, irrigation used, and drainage established. If the uterus and adnexa become thoroughly infected, they should be extirpated.

284 Franklin Street.

